# The role of neuro-ophthalmology in post-operative follow-up after resection of non-functioning pituitary macroadenomas in the UK: a clinician practice survey

**DOI:** 10.1038/s41433-026-04628-5

**Published:** 2026-09-11

**Authors:** Peter Rae, Fion Bremner, Hani Marcus, Zhaleh Khaleeli, Victoria A. Nowak, Citable collaborators, Wendy Adams, Nadeem Ali, Lidia Alvarez, Samira Anwar, Denize Atan, Shveta Bansal, Victoria Barrett, Adam Bates, James Benzimra, Tasanee Braithwaite, Michael Burdon, Ailbhe Burke, Margaret Dayan, Simon Epps, Simon Hickman, James McHugh, Helena Lee, Eduardo Miranda, Susie Mollan, Eoin O’ Sullivan, Axel Petzold, Srilakshmi Sharma, Shery Thomas, Mandagere Vishwanath

**Affiliations:** 1 North Middlesex University Hospital, London, UK; 2 National Hospital for Neurology and Neurosurgery, London, UK; 3 Somerset NHS Foundation Trust, London, UK; 4 South Tyneside and Sunderland NHS Foundation Trust, Sunderland, UK; 5 St George’s University Hospitals NHS Foundation Trust, London, UK; 6 Sandwell and West Birmingham Hospitals NHS Trust, Birmingham, UK; 7 University Hospitals of Leicester NHS Trust, Leicester, UK; 8 University Hospitals Bristol NHS Foundation Trust, Bristol, UK; 9 Lancashire Teaching Hospitals NHS Trust, Preston, UK; 10 Brighton and Sussex University Hospitals NHS Trust, Brighton, UK; 11 Maidstone and Tunbridge Wells NHS Trust, Maidstone, UK; 12 Royal Devon University Healthcare NHS Foundation Trust, Exeter, UK; 13 Guy’s and St Thomas’ Hospital NHS Foundation Trust, London, UK; 14 University Hospitals Birmingham NHS Foundation Trust, Birmingham, UK; 15 Moorfields Eye Hospital NHS Foundation Trust, London, UK; 16 Newcastle Hospitals NHS Foundation Trust, Newcastle, UK; 17 Sheffield Teaching Hospitals NHS Foundation Trust, Sheffield, UK; 18 King’s College Hospital NHS Foundation Trust, London, UK; 19 University Hospital Southampton NHS Foundation Trust, Southampton, UK; 20 Oxford University Hospitals NHS Foundation Trust, Oxford, UK; 21 Nottingham University Hospitals NHS Foundation Trust, Nottingham, UK; 22 Manchester University NHS Foundation Trust, Manchester, UK

**Keywords:** Health services, Health care

Pituitary adenomas are managed by a multi-disciplinary team (MDT) [1, 2]. The main treatment for visually-symptomatic, non-functioning macroadenomas is surgery [3]. There are currently no national guidelines regarding ophthalmology follow-up. We asked neuro-ophthalmology consultants in the UK about post-operative monitoring. A pilot survey was sent and questions refined before a second survey was distributed to all members of the British and Irish Neuro-Ophthalmology Club (BINOC), a group of UK neuro-ophthalmologists (May–August 2025). We asked about monitoring modalities, clinic type, follow-up and discharge. Fig. 1.

**Fig. 1:**
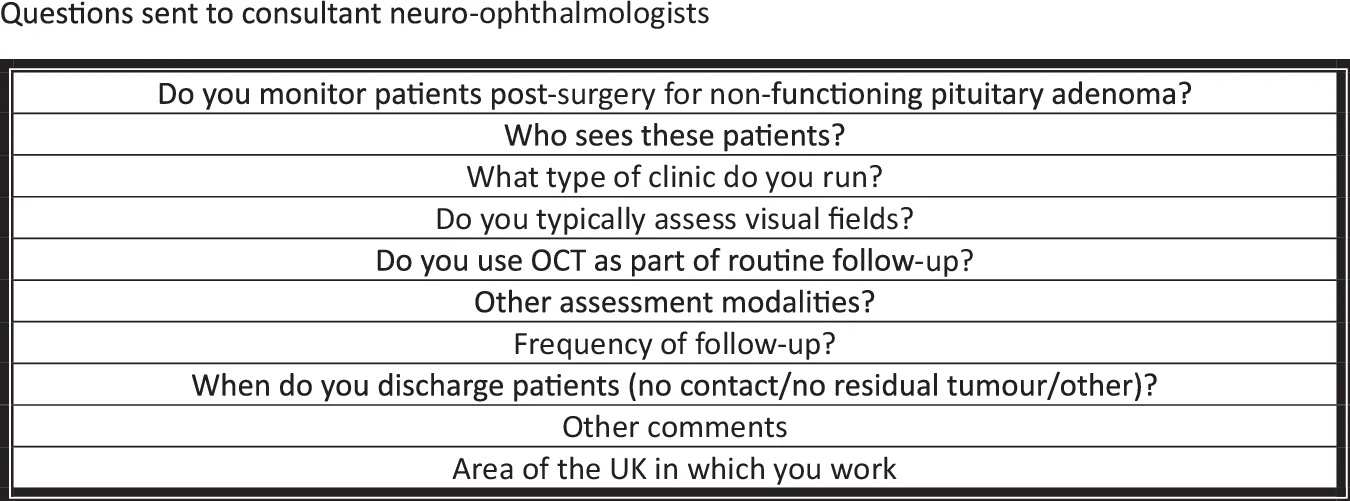
Questions sent to consultant neuro-ophthalmologists.

There were 36 responses, representing a response rate of 36% (Supplementary Table 1). There were 4 responses from North East England, 4 from the North West, 7 from the Midlands, 4 from the South West and Wales, 6 from the South East, and 11 from London. 34/36 (94%) felt neuro-ophthalmologists have a role in monitoring patients post-operatively. 11 (33%) run a ‘technician’ clinic, with data subsequently reviewed by the consultant, 10 (29%) see patients in a consultant clinic, and 13 (38%) run combined clinics.

All involved in monitoring perform visual field testing. 10 (29%) perform Goldmann visual fields, and 24 (71%) perform Humphrey visual fields, with 5 (21%) specifying 30-2, and 13 (54%) 24-2. 4 participants (12%) perform Octopus visual fields, and 2 (6%) assess fields by confrontation. Some respondents use one method or more, commenting that it would depend on the patient.

33 respondents (97%) perform OCT. Of these, 28 (85%) would scan macula and disc, whilst 5 (15%) did not specify. 33 respondents (97%) measure visual acuity. Most checked color vision (27 participants, 79%) and pupils (18 participants, 53%), with fewer assessing motility (12 participants, 35%) and funduscopy (10 participants, 29%).

10 participants (29%) said follow-up frequency would depend on patient factors. 8 (23%) initially monitor 3-monthly, then increase the interval to annually. 7 (21%) monitor annually as routine. 5 (15%) initially monitor 6-monthly, then reduce frequency to 9-monthly. 2 (6%) monitor patients more than 6-monthly, longer term. 2 (6%) see patients once before discharging to community services.

4 participants (12%) discharge patients once there is no tumour in contact with the optic chiasm, 6 (18%) when there is no residual tumour on imaging and 9 (26%) rely on the advice of the MDT. 9 (26%) discharge patients when the tumour is stable on imaging, over 1 to 10 years. 2 (6%) typically discharge after a first post-operative review in the neuro-ophthalmology clinic. 4 participants (12%) do not typically discharge patients from their clinic.

We have identified significant heterogeneity in current practice: while most neuro-ophthalmologists are involved in post-operative monitoring, decisions about discharge are particularly variable. Our survey respondents may not reflect the practice of all ophthalmologists looking after this patient group and may not have accurately described what is done in the clinic. Results may have underestimated true variability. Reasons for heterogeneity identified could be due to a lack of evidence about optimal management and reflect equipment availability and resource limitations.

Neuro-ophthalmologists can provide valuable information to inform decision-making about further treatment [4]. Standardising practice could optimise treatment and lead to better patient outcomes. A systematic review would be a helpful next step, which may provide enough information to develop robust guidelines.

## Supplementary information

Table 1 (Supplementary Figure)

## Data Availability

All data analysed during this study are included in this published article and its supplementary information files.
