# Supplementary material for: The role of neuro-ophthalmology in post-operative follow-up after resection of non-functioning pituitary macroadenomas in the UK: a clinician practice survey
Source: Eye (Lond). 2026 Sep 11;40(16):2666–7. doi: 10.1038/s41433-026-04628-5 (PMC13754796; doi:10.1038/s41433-026-04628-5)
Supplement: Supplementary file 1 [file 41433_2026_4628_MOESM1_ESM.docx]

Table 1 (Supplementary figure) Results

| **_Response Number_** | **_Involved?_** | **_Who?_** | **_F2F/Virtual?_** | **_Method of fields_** | **_OCT_** | **_Other modalities_** | **_Frequency_** | **_Frequency comments_** | **_Discharge_** | **_Discharge comments_** |
| --- | --- | --- | --- | --- | --- | --- | --- | --- | --- | --- |
| **_Pilot survey responses_** | | | | | | | | | | |
| _1_ | _N_ | _Orthoptist, Endocrine team_ | _N/A_ | _Goldmann_ | _N/A_ | _N/A_ | _N/A_ |  | _N/A_ |  |
| _2_ | _Y_ | _Ophthalmologist_ | _F2F_ | _Goldmann, Humphrey_ | _Mac/disc_ | _Colour vision, IOP, Visual acuity_ | _Other_ | _3m, gradually reduced to annually_ | _Never_ |  |
| _3_ | _Y_ | _Orthoptist_ | _Virtual_ | _Humphrey 30-2_ | _Mac/disc_ | _Visual acuity_ | _Other_ | _Once only_ | _Other_ | _To community optometry services after first appointment_ |
| _4_ | _Y_ | _Orthoptist_ | _Virtual_ | _Humphrey_ | _N/A_ | _N/A_ | _Other_ | _Depends entirely_ | _Other_ | _Depends on MDT_ |
| _5_ | _Y_ | _Ophthalmologist_ | _F2F_ | _VF (not specified)_ | _Mac/disc_ | _Colour vision. Pupils, Visual acuity_ | _4m_ |  | _Other_ | _Stable appearances 4 months apart_ |
| _6_ | _Y_ | _Ophthalmologist_ | _F2F_ | _VF (not specified)_ | _Mac/disc_ | _Colour vision, Visual acuity_ | _Other_ | _Monthly, reducing to 4m_ | _No residual_ |  |
| _7_ | _Y_ | _Ophthalmologist, orthoptist_ | _Both_ | _VF (not specified)_ | _Mac/disc_ | _Colour vision, IOP, Ocular motility, Pupils, Visual acuity_ | _12m_ |  | _No contact_ |  |
| _8_ | _N_ | _Endocrine team only_ | _N/A_ | _N/A_ | _N/A_ | _N/A_ | _N/A_ |  | _N/A_ |  |
| _9_ | _Y_ | _Ophthalmologist, orthoptist_ | _Both_ | _Humphrey_ | _OCT_ | _Colour vision, Visual acuity_ | _Other_ | _3m, reducing to 6-9m_ | _Other_ | _Depends on MDT_ |
| _10_ | _Y_ | _Ophthalmologist_ | _F2F_ | _Humphrey_ | _Mac/disc_ | _Colour vision, Ocular motility, Pupils, Visual acuity_ | _12m_ |  | _Never_ |  |
| _11_ | _Y_ | _Neurologist trained in neuro-ophthalmology_ | _F2F_ | _Humphrey_ | _OCT_ | _Colour vision, Slit lamp, Visual acuity_ | _Other_ | _Early post-op, reducing gradually to 3m, 6m, 12m, 24m_ | _Never_ |  |
| _12_ | _Y_ | _Ophthalmologist_ | _F2F_ | _Humphrey_ | _OCT_ | _Visual Acuity_ | _12m_ | _Partly depends_ | _Other_ | _Depends on MDT_ |
| _13_ | _Y_ | _Orthoptist_ | _Virtual_ | _VF (not specified)_ | _OCT_ | _Colour vision, Slit lamp, Visual acuity_ | _12m_ |  | _Other_ | _Depends on MDT_ |
| _14_ | _Y_ | _Ophthalmologist, Optician_ | _Both_ | _VF (not specified)_ | _OCT_ | _Colour vision, Ocular motility, Pupils, Visual acuity_ | _Other_ | _Once only_ | _Other_ | _To community optometry services after first appointment_ |
| **_UKNOS/BINOC survey responses_** | | | | | | | | | | |
| _1_ | _Y_ | _Ophthalmologist, Orthoptist_ | _Virtual_ | _Goldmann_ | _Mac/disc_ | _Colour vision, Visual acuity_ | _Other_ | _3m, 9m, then depends on imaging_ | _No contact_ |  |
| _2_ | _Y_ | _Ophthalmologist, Orthoptist_ | _Both_ | _Humphrey 30-2_ | _Mac/disc_ | _Other, Visual acuity, IOP_ | _Other_ | _3m then depends on imaging_ | _Other_ | _Imaging/residual/stability_ |
| _3_ | _Y_ | _Orthoptist_ | _Virtual_ | _Humphrey 30-2_ | _Mac/disc_ | _Colour vision, Pupils, Visual acuity_ | _6m_ | _6m then 12m_ | _No residual_ |  |
| _4_ | _Y_ | _Ophthalmologist_ | _Both_ | _Humphrey 24-2_ | _Mac/disc_ | _Slit lamp, Visual acuity_ | _Other_ | _3m then 12m when stable_ | _Other_ | _VF and imaging stable_ |
| _5_ | _Y_ | _Orthoptist_ | _Virtual_ | _Humphrey 24-2_ | _Mac/disc_ | _Visual acuity_ | _Other_ | _Depends entirely_ | _Other_ | _Either of the above, or stable 5 years_ |
| _6_ | _Y_ | _Ophthalmologist, Orthoptist_ | _Both_ | _Goldmann_ | _Mac/disc_ | _Colour vision, Ocular motility, Pupils, Slit lamp, Visual acuity_ | _Other_ | _Depends entirely_ | _Other_ | _Size of residual/other MDT assessment_ |
| _7_ | _Y_ | _Ophthalmologist, Orthoptist_ | _F2F_ | _Goldmann, Humphrey 24-2_ | _Mac/disc_ | _Colour vision, Ocular motility, Pupils, Slit lamp, Visual acuity_ | _Other_ | _Depends entirely_ | _No contact_ | _And no visual pathway damage_ |
| _8_ | _Y_ | _Ophthalmologist, Orthoptist_ | _Both_ | _Humphrey 24-2_ | _Mac/disc_ | _Visual acuity_ | _Other_ | _Depends entirely_ | _No residual_ |  |
| _9_ | _Y_ | _Ophthalmologist, Orthoptist_ | _F2F_ | _Humphrey 24-2_ | _Mac/disc_ | _Colour vision, Visual acuity_ | _6m_ | _Partly depends_ | _No residual_ |  |
| _10_ | _Y_ | _Orthoptist_ | _Virtual_ | _Humphrey 24-2_ | _Mac/disc_ | _Colour vision, Ocular motility, Pupils, Visual acuity_ | _Other_ | _6-9m_ | _Other_ | _Advised by MDT_ |
| _11_ | _Y_ | _Ophthalmologist_ | _Both_ | _Goldmann, Humphrey 24-2_ | _Mac/disc_ | _Colour vision, Pupils, Slit lamp, Visual acuity_ | _Other_ | _Depends entirely_ | _No contact_ | _If other follow up in place_ |
| _12_ | _Y_ | _Ophthalmologist, Orthoptist_ | _Virtual_ | _Octopus_ | _Mac/disc_ | _Colour vision, Ocular motility, Pupils, Visual acuity_ | _Other_ | _Depends – 18m when stable_ | _No residual_ |  |
| _13_ | _Y_ | _Neurologist trained in neuro-ophthalmology_ | _Both_ | _Confrontation, Goldmann, Humphrey 24-2_ | _Mac/disc_ | _Colour vision, Visual acuity_ | _12m_ |  | _Other_ | _Stable over prolonged follow-up_ |
| _14_ | _Y_ | _Ophthalmologist, Orthoptist_ | _Both_ | _Humphrey 30-2, Octopus_ | _Mac/disc_ | _Colour vision, Pupils, Visual acuity_ | _6m_ |  | _Other_ | _Do not typically discharge_ |
| _15_ | _Y_ | _Ophthalmologist, Orthoptist_ | _Both_ | _Goldmann_ | _Mac/disc_ | _Colour vision, Ocular motility, Pupils, Slit lamp, Visual acuity_ | _Other_ | _Depends entirely_ | _Other_ | _Advised by MDT_ |
| _16_ | _Y_ | _Ophthalmologist_ | _F2F_ | _Humphrey 24-2_ | _Mac/disc_ | _Colour vision, Pupils, Slit lamp, Visual acuity_ | _Other_ | _Depends entirely_ | _Other_ | _Advised by MDT_ |
| _17_ | _Y_ | _Orthoptist_ | _Virtual_ | _Octopus_ | _Mac/disc_ | _Colour vision, Ocular motility, Pupils, Visual acuity_ | _3m_ | _3m reducing to 6m then 9m_ | _Other_ | _No residual / stable for 10 years_ |
| _18_ | _Y_ | _Ophthalmologist, Other_ | _Both_ | _Humphrey 24-2_ | _Mac/disc_ | _Colour vision, Visual acuity_ | _Other_ | _Usually 12m_ | _No residual_ |  |
| _19_ | _Y_ | _Orthoptist_ | _Virtual_ | _Confrontation, Humphrey 30-2, Octopus_ | _Mac/disc_ | _Colour vision, Ocular motility, Pupils, Visual acuity_ | _Other_ | _3 m reducing to 6m then 12m_ | _Other_ | _No residual and stable for 5 years_ |
| _20_ | _Y_ | _Orthoptist_ | _Virtual_ | _Goldmann, Humphrey 24-2_ | _Mac/disc_ | _Colour vision, Ocular motility, Pupils, Visual acuity_ | _Other_ | _3m or 6m_ | _Other_ | _No contact, stable 1 year and community services available_ |
| _21_ | _Y_ | _Ophthalmologist_ | _Both_ | _Humphrey 24-2_ | _Mac/disc_ | _Colour vision, Ocular motility, Pupils, Slit lamp, Visual acuity_ | _Other_ | _Depends entirely_ | _Other_ | _No residual, stable for 2+ years_ |
| _22_ | _Y_ | _Ophthalmologist, Orthoptist_ | _F2F_ | _Goldmann, Humphrey 24-2_ | _Mac/disc_ | _Colour vision, Pupils, Slit lamp, Visual acuity_ | _12m_ | _May be more often_ | _Other_ | _Advised by MDT_ |
